# Systematic characterization of the effect of Ag@TiO_2_ nanoparticles on the performance of plasmonic dye-sensitized solar cells

**DOI:** 10.1038/s41598-017-15541-z

**Published:** 2017-11-16

**Authors:** Pascal Nbelayim, Go Kawamura, Wai Kian Tan, Hiroyuki Muto, Atsunori Matsuda

**Affiliations:** 1 Dept. of Electrical and Electronic Info. Eng., Grad. Sch. of Eng., Toyohashi University of Technology, 1-1 Hibarigaoka, Tempaku-cho, Toyohashi, Aichi 441-8580 Japan; 2 Center for International Education, Toyohashi University of Technology, 1-1 Hibarigaoka, Tempaku-cho, Toyohashi, Aichi 441-8580 Japan; 3 Institute of Liberal Arts and Sciences, Toyohashi University of Technology, 1-1 Hibarigaoka, Tempaku-cho, Toyohashi, Aichi 441-8580 Japan

## Abstract

The use of plasmonic nanoparticles (NPs) in dye-sensitized solar cells (DSSCs) in an effort to enhance their power conversion efficiencies (PCEs) increases light absorbance of the cells but also affect their electron dynamics. This has contributed to the failure of plasmonic NPs to make the expected high impact of PCE enhancement. Herein, we investigated the wide range effects of plasmonic NPs on the performance of DSSCs, using extended characterization and a systematic approach. We prepared DSSCs using Ag@TiO_2_ NPs-doped TiO_2_ photoanodes. Using a wide range doping concentration, we obtained panchromatic enhancement effect with two optimal doping concentrations (0.1 and 1 wt. %).They enhanced PCE via mainly: a) optimal band alignment for efficient charge injection; and b) a balance of the negative and positive effects of plasmonic NPs on cell performance parameters (open circuit voltage, fill factor, charge transfer resistance against recombination, electron life time and charge collection efficiency); respectively. The PCE of the pristine sample increased from 4.66 to 4.88 and 5.00% via these 2 routes, respectively. The major cause of not obtaining very high PCE was charge recombination from high charge density. Thus, these observations might serve as invaluable guidance for the preparation of highly efficient plasmonic DSSCs.

## Introduction

Dye-sensitized solar cells (DSSCs) have evolved as credible alternative to conventional solid state p-n junction photovoltaics, with their core advantages of facile & low-cost fabrication, environmentally friendly, and good performance under lowlight conditions^1–4^. The highest power conversion efficiency (PCEs) of the DSSC is between 11 and 13%^5,6^, since its research boom in 1991, when M. Gratzel & B. Oregon obtained an unprecedented PCE of about 7%, using a nanoporous TiO_2_ photoanode^7^. Although groups like G24, Sony and Aisin Seiki have commercialised DSSC for niche markets^8,9^, however, an estimated PCE ≥ 20% is necessary for commercial high-power applications and utility-scale power generation^2^. A key factor to increase the PCE is to increase the light absorbance of the photoanode of the DSSC. This approach includes designs such as: large surface area mesoporous photoanodes; hierarchically nano-structured & scattering top-layer photoanodes; design of new panchromatic absorbing dyes; photonic crystal photoanodes and plasmonic photoanodes^10^. Among these, the plasmonic photoanodes have the critical advantages of: a) facile preparation; b) use of less photoanode materials; and c) the high potential of extremely enhanced light absorbance via the localized surface plasmon resonance (LSPR) effect^2,3,11^, through radiative and non-radiative mechanisms of plasmon energy transfer^2^. However, the highest enhanced plasmonic DSSC PCE is still < 11%^2^. This is mainly because of the secondary effects of plasmonic nano-structures on the electron dynamics of the DSSC, apart from their light absorbance enhancement effect. Thus, the need for the understanding of these secondary effects for the optimal application of plasmonic nano-structures to significantly increase the PCE of the DSSC.

Villanueva-Cab *et al*.^12^. investigated the assumption that bare plasmonic metal NPs work as recombination centres in plasmonic DSSCs with the intention to improve the lack of clear understanding of the charge transfer kinetics in plasmonic DSSCs. They used 3.6 µm-thick TiO_2_ photoanodes with different amounts of ~2 mM colloidal Au (~20 nm) solution impregnation (Au1, Au4 & Au5; which are 1, 4 and 5 rounds of 20 µL of the colloidal Au treatments, respectively). They observed that the dyed photoanodes recorded lower light absorbances than the reference, except for Au1, in the visible region, but recorded higher absorbances in the near-infrared region. Their current-voltage (I-V) characterization showed lower and decreasing short-circuit current density (*J*_*sc*_) and lower open-circuit voltage (*V*_*oc*_) than the reference, although Au5 showed higher values than Au4. Small perturbation measurements showed lower and decreasing recombination constant, charge transfer resistance (*R*_*ct*_) and electron life time (*τ*_*n*_) than the reference sample. Charge collection efficiency (*η*_*cc*_) was said to be constant. They concluded that Au NPs acting as recombination centres cannot fully explain photovoltaic behaviours of DSSCs; and that variations in *J*_*sc*_ and *V*_*oc*_ were caused by decreasing charge injection efficiency (*η*_*inj*_) due to upward conduction band (CB) shift in TiO_2_ from the effect of the Au NPs.

Choi *et al*.^13^. reported that plasmonic NPs in DSSCs have two effects: the plasmonic (light absorbance enhancement) and charging (electron dynamics) effects. They used Au NPs with two different cappings: SiO_2_ (an insulator to prevent electron charging of the Au core) and TiO_2_ (a semiconductor that would allow transfer of electrons to charge the Au core). They observed enhanced absorbance with a consequent increase in *J*_*sc*_ in both samples, with accompanying increase in *V*_*oc*_ in the TiO_2_-capped sample. There was no significant change in *V*_*oc*_ in the SiO_2_-capped sample, although it recorded the higher *J*_*sc*_. The increase in *V*_*oc*_ was the result of the Au NPs accepting electrons from TiO_2_ shell and TiO_2_/dye and undergoing fermi level equilibration (charging effect). They also studied the plasmonic effects on the cell performance parameters with five loading concentrations (0.2–1%). Their results showed no significant effect on fill factor (*FF*) but an effect of decreasing *J*_*sc*_ at loading concentrations >0.7%, which they suggest was probably due to filtering effects of the Au core.

Qi *et al*.^14^. observed the enhancement effect on light absorbance, and a resultant enhanced *J*_*sc*_ and PCE. They observed the major plasmonic NPs enhancement effect on incident photon-to-current efficiency (IPCE) in the wavelength region of 400–500 nm, where the LSPR peak of their NPs is located. They were also able to reduce photoanode thickness and still obtained a higher PCE. They did not observe any significant difference between the *FF* and *V*_*oc*_ values of the pristine and plasmonic DSSCs. They however, observed decreased *J*_*sc*_ and PCE at higher (>0.6%) plasmonic doping conc., which they suggested was probably due to increased trapping of photogenerated electrons by Ag and conversion of part of the incident solar power into heat.

In our laboratory, using bare Ag NPs-filled TiO_2_ nanotube plasmonic DSSCs, fabricated with various growth techniques and NPs-loading approaches^15–17^, we observed enhanced light absorption, *J*_*sc*_ and PCE. We also observed increased *V*_*oc*_ and *FF*, but in one study with electrochemical impedance spectroscopy (EIS) evaluation, we observed a reduction in *R*_*ct*_ and *τ*_*n*_^15^.

From the above review, there are significant variations in these reported results on the effects of plasmonic NPs in DSSCs, barring the differences in the DSSC types employed; and somewhat inconclusive results, suggesting the need for more systematic studies on the effects of plasmonic NPs on the performance of DSSCs. We think this is because of the use of limited characterizations with fewer and short-range doping plasmonic concentrations.

In this work we have used extended characterizations (optical absorption; photoluminescence spectroscopy (PL); I-V; EIS and IPCE); and wider range of plasmonic NPs doping concentrations (0.1, 0.25, 0.5, 1 and 5%), offering a high dynamic range. In addition, we employed a systematic approach, to obtain a more in-depth understanding of the effects of plasmonic NPs on the performance DSSCs, in addition to the synergistic effect of these effects. We used Ag@TiO_2_ core-shell (C-S) NPs-doped TiO_2_ pastes and a pristine un-doped paste as a reference, for the fabrication of our photoanodes/DSSCs. We observed two optimally enhanced PCEs: at 0.1 and 1% doping concentrations, with the dominant plasmonic enhancement effects of optimal band alignment for efficient charge injection on one hand; and an optimal balance of the charge carrier intercalation/dynamics on the other hand, respectively. We also observed some various general effect-trends of the plasmonic NPs on the various cell performance parameters.

## Results and Discussion

### Morphology and composition of Ag@TiO_2_ C-S NPs

**Figure 1:**
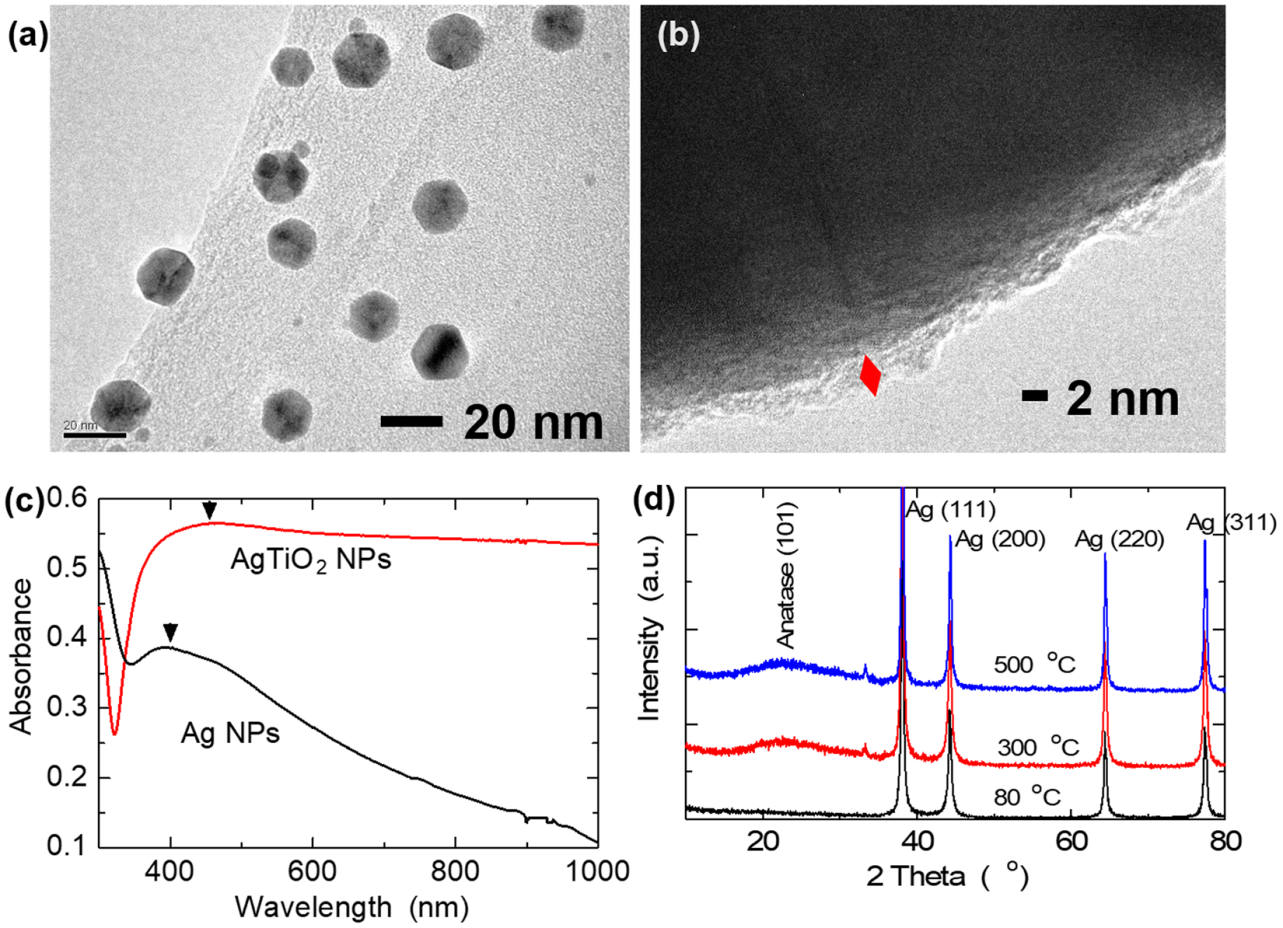
(**a**) TEM image of Ag@TiO_2_ NPs after 350 °C HT. (**b**) HRTEM of the Ag@TiO_2_ NPs showing the thin TiO_2_ shell. (**c**) Optical absorption spectra of Ag and Ag@TiO_2_ NPs showing a red-shift in the LSPR peak position, indicating the formation of the C-S structure. (**d**) XRD patterns of the Ag@TiO_2_ NPs at various HT temperatures showing an amorphous shell, as prepared, and crystallization into the anatase phase after 350 °C.

We synthesized the Ag@TiO_2_ NPs via a sol process, using Pluronic P-123 as a dispersing agent. Figure 1a. shows the TEM image of uniformly sized NPs of about 20 nm, after 350 °C heat treatment (HT). The targeted 20 nm particle size achieved was to closely match that of the main photoanode material of TiO_2_. This was to minimize the effect of doping NPs on the surface area of the mesoporous photoanodes. Thermal analysis of the C-S NPs gel and that of Pluronic P-123 showed combustion of the Pluronic P-123 dispersant at ~ 264 °C, and hence, the HT at 350 °C to obtain the pure Ag@TiO_2_ C-S NPs. Figure 1(b) is the HRTEM image showing the TiO_2_ shell with an avg. thickness of ~ 2 nm, which is essential for the protection of the plasmonic Ag core from the high temperature processing of the photoanode; corrosive effect of the cell electrolyte; as well as allow for the transmittance of the LSPR effect of the Ag core to vicinal dye and TiO_2_ NPs.

To investigate the formation of the plasmonic Ag and Ag@TiO_2_ NPs, we studied the optical absorption characteristics of these NPs, Fig. 1c. We observed the characteristic red-shift in LSPR peak position, from 404 nm of the Ag NPs to an estimated peak at 472 nm of the broadband spectrum of the Ag@TiO_2_ NPs. The peak shift and enhanced absorbance, with extended absorbance into the near-infrared (NIR) region, are attributed to the higher refractive index of the TiO_2_ shell with undulating structure. This anisotropic shell structure would increase polarizability, ultimately resulting in different LSPR modes and multiple plasmon resonances^2^. Typically, a broadband light absorbance is achieved by using bimetallic or rod-like structures^2,18^. However, we have achieved this using a single capped metal nanosphere. We investigated the composition and crystalline structure of our C-S NPs using X-ray diffraction (XRD) characterization. The as-prepared C-S NPs after the first HT at 80 °C showed diffraction patterns of cubic Ag (111), (200), (220) & (311) planes (JCPDS no. 04–0783), Fig. 1(d). There were no observed TiO_2_ peaks, suggesting an amorphous TiO_2_ shell in the as-prepared C-S NPs. At 350 °C HT and beyond, the diffraction pattern from the (101) and (004) planes of anatase phase TiO_2_ were observed, with additional peaks of the (103) and (112) planes observed after 500 °C HT (JCPDS no. 21–1272), Fig. 1d. The broad (101) anatase plane peak is attributed to the small crystallite size and/or crystalline strain.

### Corrosion stability and optical enhancement effect of the plasmonic Ag@TiO_2_ NPs

**Figure 2:**
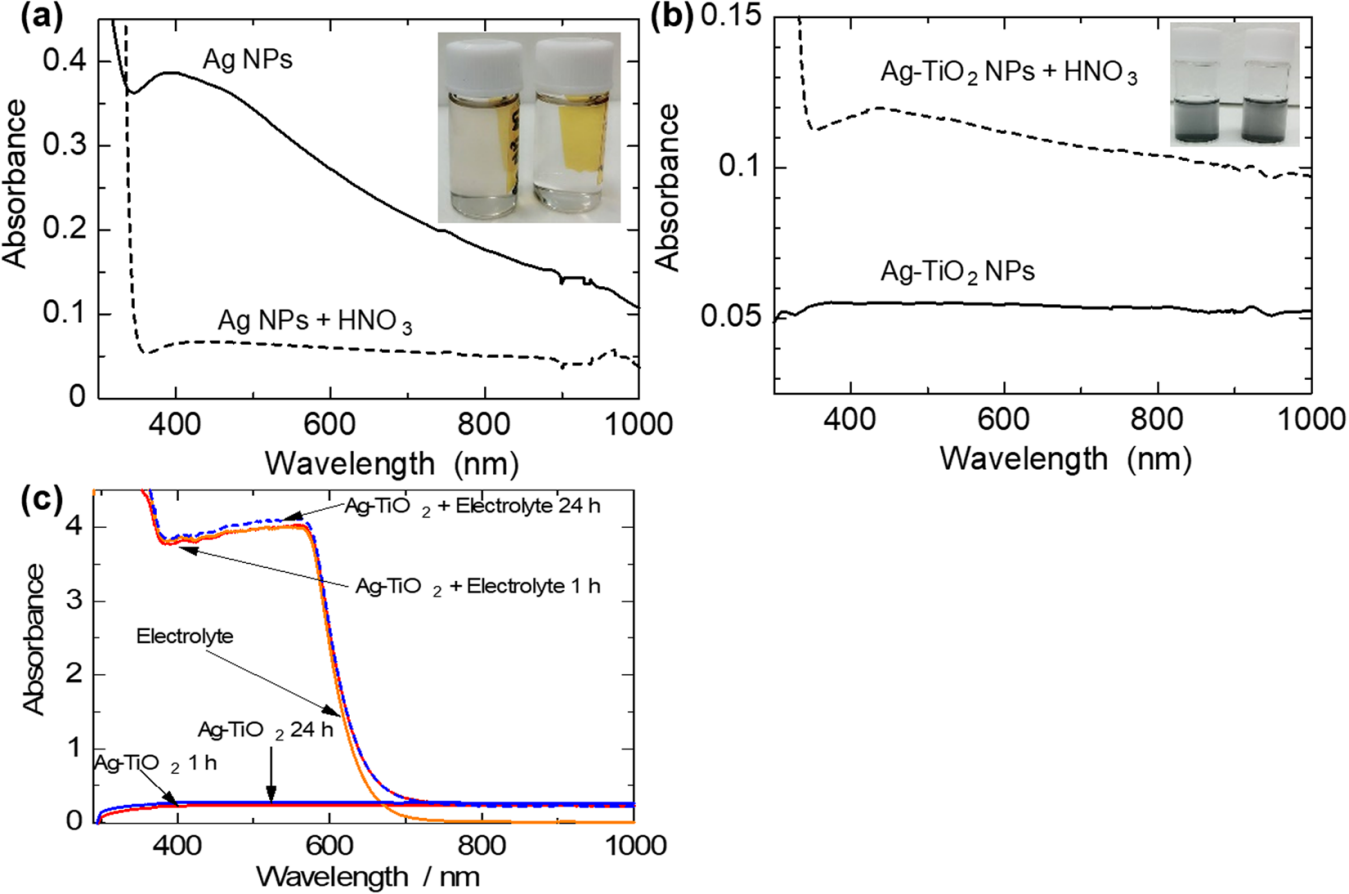
(**a**) Optical absorption spectra showing the dissolution of Ag NPs in acid; inset is a visual image of the test samples; without acid (left) & with acid (right) treatment. (**b**) Optical absorption spectra showing corrosion stability test of Ag@TiO_2_ NPs via acid treatment; inset is a visual image of the test samples; without acid (left) & after acid (right) treatments. (**c**) Absorbance spectra showing stability of the Ag@TiO_2_ C-S PNPs in I^−^/I_3_^−^ electrolyte.

One of the functions of the TiO_2_ shell is to protect the Ag core from the corrosive effect of the I^-^/I_3_^-^ electrolyte. To investigate this, we performed an acid stability test via optical absorbance spectroscopy. The results are shown in Fig. 2(a,b). Comparing the absorbance spectra of the Ag NPs solutions in Fig. 2(a), the LSPR peak disappears in the spectrum of the acid treated sample, with accompanying drastic decrease in absorbance intensity, indicating the dissolution of the Ag NPs. This is confirmed visually in the inset image, which shows a light brown colloidal solution of the Ag NPs, of the sample without acid treatment (left), but a clear solution of the acid treated sample (right). However, in the case of the Ag@TiO_2_ NPs, the inset image of Fig. 2(b), there is no observed difference between the sample without acid treatment (left) and the acid-treated sample (right).

Comparing their optical spectra, there is no loss of absorbance intensity in the acid-treated sample, indicating that the TiO_2_ shell protects the Ag core from corrosive effects. The apparent increase in absorbance in the acid-treated sample is probably due to a dilution effect of the acid treatment causing a constructive interaction effect of the different plasmon modes^19^. To further consolidate these results we also tested the stability of the C-S NPs in the I^-^/I_3_^-^ electrolyte (2.2 mg PNPs in 3 mL electrolyte). The results are shown in Fig. 2(c), with spectra of the electrolyte and C-S PNPs only (2.2 mg in 3 mL acetonitrile (solvent for the electrolyte)) for comparison. As can be seen, there is no significant change in absorbance of the C-S PNPs-electrolyte mixture spectrum, even after 24 h, especially in the NIR region where the spectrum of the mixture matches that of the C-S NPs only. That is, if there were dissolution of the PNPs, the absorbance of the mixture would have been lower than the spectrum of the C-S NPs only.

**Figure 3:**
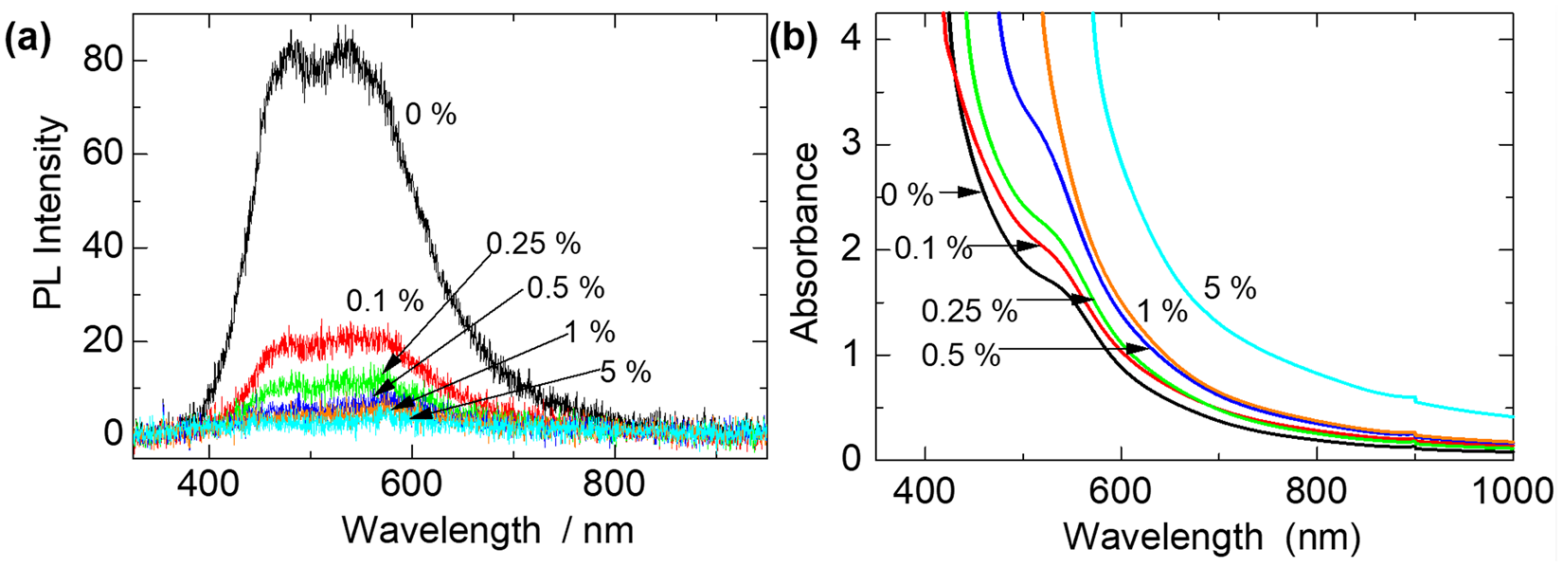
(**a**) PL spectra of photoanodes showing the electron-sink effect via decreasing PL intensity with increasing amount of Ag@TiO_2_ NPs doping. (**b**) Optical absorption spectra showing enhanced broadband light absorbance in the Ag@TiO_2_-doped photoanodes.

Recombination of photogenerated electrons during their transport within the mesoporous nanocrystalline TiO_2_ network of DSSCs is one of the major factors that contributes to the inefficient performance of DSSCs. Metalic nanoparticles have been reported to exhibit electron-sink (or photocharging) effect^13,20–22^, and thus, the potential to minimize charge recombination. A suitable technique for investigating this electron-sink effect is PL spectroscopy, which studies photon emissions resulting from electron-hole recombination. Anatase is an indirect band gap material and hence, should not exhibit PL. However, it has intrinsic oxygen vacancies (responsible for its n-type semiconducting properties)^22^, which generate different kinds of electron and hole traps, randomly distributed within its forbidden band gap. This creates various transitions, resulting in the typical broadband visible PL spectrum of TiO_2_^23,24^, as observed in our results in Fig. 3(a).

The generally low PL intensity observed in our samples is attributed to the mesoporous and thin film (6.5 μm) characteristics of our samples, as reported by Mercado *et al*.^25^. In Fig. 3(a) the PL intensity decreases with increasing amount of Ag@TiO_2_ doping, indicating that the excited electrons are captured by the core Ag NPs before they recombine, causing the so called electron-sink effect. This causes the accumulation of electrons on the Ag NPs, which improves electron life time and can thus, reduce charge recombination, ultimately, improving electron transport.

To investigate the LSPR effect on the light absorption enhancement of dye molecules of the DSSC photoanode, we measured the optical absorbance of dye-sensitized plasmonic photoanodes and compared them to that of the reference un-doped photoanode. The results are shown in Fig. 3(b). The plasmonic photoanodes recorded higher absorbances than the pristine photoanode. This is a typical result in many reported works on plasmonic DSSCs. The absorbance increased with increasing amount of doping, with an analogous increasing effect in the NIR region. All photoanodes were of the same average thickness of 6.6 ± 0.1 µm. Thus, the increasing absorbance could only be attributed to the LSPR effect of the plasmonic NPs.

**Figure 4:**
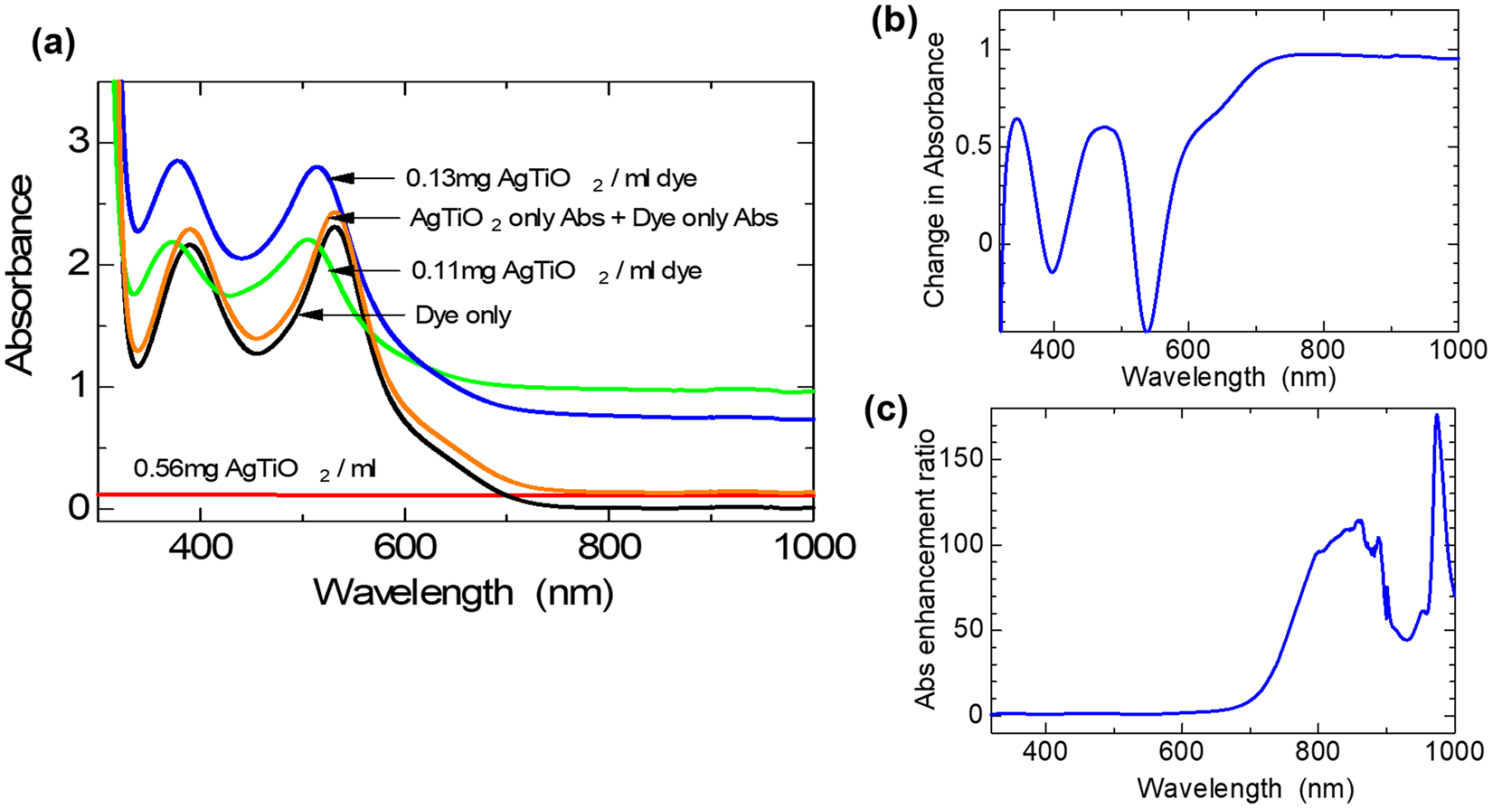
Optical absorption spectra showing enhanced broadband light absorbance of the N719 dye: (**a**) separate dye and plasmonic NPs; dye plus plasmonic NPs mixtures; and arithmetic addition of dye and plasmonic NPs absorbances. (**b**) Absorbance of [0.12 mg ml^−1^ dye] – absorbance of [dye-only]. (**c**) Absorbance of [0.12 mg ml^−1^ dye] ÷ absorbance of [dye-only].

To further investigate the light absorbance enhancement effect of the plasmonic NPs on the dye, we studied the optical absorption patterns of various dye-plasmonic NPs compositions in solution. This was to simulate the plasmonic enhancement effect of the PNPs on the dye without the possible interference effects of other components of the photoanode or complete DSSC. The results are shown in Fig. 4. As can be observed in Fig. 4(a), the plasmonic NPs-spiked dye solutions (green and blue spectra) both showed enhanced light absorption over the pristine dye and plasmonic NPs (black and red spectra, respectively).

The quantum of enhancement further increased with the increase in amount of plasmonic NPs, indicating the LSPR light absorption enhancing effect on the dye.

We also studied the synergistic effect of the dye-plasmonic NPs in the enhancement by comparing the absorption spectra of an arithmetic addition of the separate absorbances of the pristine dye and plasmonic NPs on one hand (orange spectrum), to the absorption spectra of the plasmonic NPs-spiked dye solutions. The spectra of the spiked dye solutions matched each other, with blue-shift in absorption peaks, compared to the pristine dye, whiles they differed from the spectrum of the arithmetic sum (orange spectrum), which matched that of the pristine dye. This spectrum also showed lower absorbance than the spiked dye spectra, although it had much higher plasmonic NPs concentration. These results show a synergistic effect between the dye and plasmonic NPs, on the light absorption enhancement effect. This peak shift has been observed in the reported works of Qi *et al*.^14^. and Choi *et al*.^13^. Whiles Qi *et al*. studied the enhanced absorbance with respect to time, where they observed increasing absorbance over time, but without further peak shift. They did not explicitly state the cause of the peak shift, but attributed the enhanced absorbance over time to increasing layering of the dye molecules on plasmonic NPs, with consequent increase in proximity of layered dye molecules to neighbouring plasmonic NPs. Choi *et al*. attributed their observed peak shift to excitation of the TiO_2_ semiconductor shell. Their results showed no peak shift in SiO_2_-capped Au NPs (SiO_2_ is an insulator) but a continuous peak shifting, in the TiO_2_-capped Au NPs (TiO_2_ is a semiconductor). Also there was no peak shift in aerated solution but peak shift in de-aerated solution. In our work, we sonicated the solutions for about 15 min and measured the optical absorbance within 30 min to1 h, storing our samples mainly in the dark. Thus, we attributed our observed peak-shift to layering of dye molecules on the plasmonic NPs, modifying the LSPR of the NPs by coupling with the plasmon and causing the observed blue-shift, analogous to the red-shift effect of TiO_2_ capping.

We also studied the effective enhancement effect of the plasmonic NPs by deducting the absorbance of the pristine dye from that of the spiked dye (Fig. 4(b)), and also dividing the absorbance of the spiked sample over that of the pristine dye (Fig. 4(c)). From Fig. 4(b) it can be observed that the enhancement effect is complementary, enhancing the dye absorbance in its poorly absorbing regions of the electromagnetic spectrum. In Fig. 4(c) a similar trend is observed, with an average of × 100 enhancement in the NIR region. Thus, our plasmonic NPs achieved a broadband optical absorption effect with the major enhancement effect in the regions where the dye exhibits weak absorbance.

### Effect of plasmonic NPs on DSSC performance

**Figure 5:**
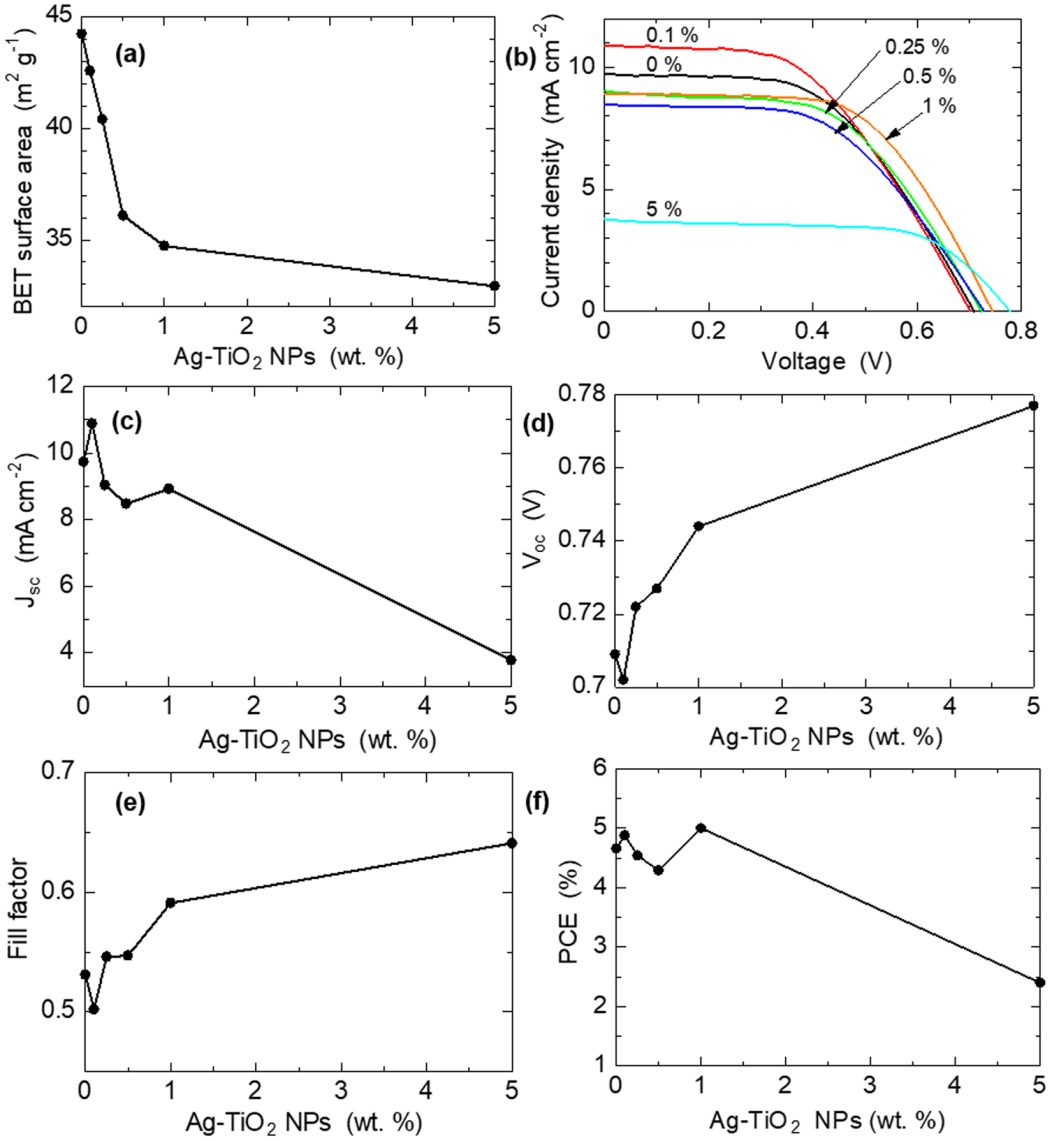
Plots of plasmonic NPs doping effects on the surface area and photovoltaic performance parameters on DSSCs: (**a**) Surface area; (**b**) I-V; (**c**) *J*_*sc*_; (**d**) *V*_*oc*_; (**e**) *FF*; (**f**) PCE.

To investigate the effect of these plasmonic NPs on the performance of the DSSC we assembled the prepared photoanodes into plasmonic DSSCs and evaluated their I-V characteristics to compare their performance to the pristine reference cell (0%), and to each other. Figure 5 shows the results of their effects and effect trends on the Brunauer–Emmett–Teller surface area (BET SA) and photovoltaic performance parameters of the cells. The first 6 columns of Table 1 is a quantitative summary of these results. From Fig. 5(a) we observe nominal and consistent decrease in SA with increasing amount of plasmonic NPs doping, from 3.7% decrease at 0.1% doping to 25.5% decrease at 5% doping. We think that this decreasing SA is due to the initially amorphous state of the TiO_2_ shell of the plasmonic NPs; such that during the heat treatment there is increased necking between the TiO_2_ shell and TiO_2_ P25 NPs, decreasing SA. This decrease in SA was expected to obviously reduce dye loading in the photoanodes, with a consequent decrease in light harvesting. However, we obtained (3.50, 3.51, 3.51, 3.53 and 3.54) * 10^–8^ moles cm^−2^ dye loadings for the photoanode samples of 0, 0.1, 0.25, 0.5, 1 and 5% plasmonic NPs loadings, respectively. Thus, the decreasing SA effect of the plasmonic NPs do not significantly affect the amount of dye absorbed for the thickness of the coated photoanodes in this work. This apparent deviation from what was expected is explained to be due to one or two reasons: 1) possible minute amount of leaching of plasmonic NPs into the soaking dye causing higher absorbance reading than should be, and a resultant false higher dye loading; 2) the modification of trap states on the surface of the TiO_2_ NPs catalyzing enhanced dye absorption. However, the verification of these explanations is beyond the scope of this work. The dye loading results were obtained via optical spectroscopy by deducting the absorbance of the 10 mL dye solution in which each photoanode was soaked from the absorbance of the dye before the soaking. A repeat of this analysis using the more common “1 mol L^−1^ NaOH dye desorption method” was also used and very similar results were obtained. Figure 5(b) is the I-V spectra of the cells, with sample 0.1% recording the highest *J*_*sc*_ and the lowest *V*_*oc*_, whiles sample 5% records the lowest *J*_*sc*_ and the highest *V*_*oc*_. Figure 5(c-d) are the extracted I-V parameters. In Fig. 5(c) we observe an increase in *J*_*sc*_ at 0.1% doping, a decrease afterwards, and then a slight increase again at 1% doping, and a return to the decreasing trend. The general observation trend in many reported plasmonic DSSC works (e.g. review work of Erwin *et al*.^2^. and works of Qi *et al*.^14^ and Lim *et al*.^22^) is an increase in *J*_*sc*_ to an optimal value and a decrease afterwards, although Ramakrishna *et al*.’s simulation work on electron injection in plasmonic metal@semiconductor C-S-dye DSSCs^37^ predicts the possibility of multiple optimal performances in charge injection.

**Table 1:** Effect of plasmonic NPs on the photoanode surface area and extracted I-V & EIS parameters.

**Sample**	**BET SA (m** ^**2**^ **g** ^**−1**^ **)**	***J***_***SC***_ **(mA cm** ^**−2**^ **)**	***V***_***OC***_ **(V)**	***FF***	**PCE (%)**	***R***_***s***_ **(Ω)**	***R***_***CT***_ **(Ω)**	***τ***_***n***_ **(ms)**	***η***_***CC***_ **/%**
**0%**	44.2	9.74	0.709	0.531	4.66	20.1	4.8	5.04	19.3
**0.1%**	42.6	10.89	0.702	0.502	4.88	20.8	4.2	4.00	16.8
**0.25%**	40.4	9.04	0.722	0.546	4.54	20.6	7.1	5.04	25.6
**0.5%**	36.1	8.48	0.727	0.547	4.29	22.1	10.4	6.34	32.0
**1%**	34.7	8.93	0.744	0.591	5.00	20.0	7.1	6.34	26.2
**5%**	32.9	3.78	0.777	0.641	2.40	21.7	15.8	12.63	42.1

The main factors that contribute to high *J*_*sc*_ is good light absorbance to generate charge carriers, their efficient injection and transport in the photoanode. From Fig. 1(d), we observed increased light absorbance of the plasmonic photoanodes over the pristine photoanode, with increasing intensity as plasmonic NPs content increased, despite their reducing SA. This indicates an overshadowing effect of the LSPR effect over the reducing SA effect. Thus, the highest *J*_*sc*_ obtained at 0.1% doping can be attributed to efficient generation, injection and transport of charge carriers. Most significantly, it reveals that the most significant enhancement is efficient charge injection, as evidenced in its reduction in *V*_*oc*_ value in Fig. 5(d) and Table 1, compared to that of the pristine sample. This is further explained as follows: there is a minimal threshold energy gap between the LUMO of a sensitizer and the CB edge of a semiconducting material, below which there is poor charge injection efficiency^26,27^. In addition, the *V*_*oc*_ can be described simply as the difference between the fermi level of the photoanode and the redox potential of the electrolyte, and hence, the observed *V*_*oc*_ change is from the plasmonic NPs-modified photoanode. Thus, at the 0.1% plasmonic NPs doping of the 6.5 µm thick TiO_2_ photoanode, there is a plasmon-photoanode material coupling that induces a lowering of the fermi level of the photoanode, to an optimal level, for efficient charge injection, that ultimately produced the highest *J*_*sc*_. For the rest of the plasmonic NPs-doped samples, we observed a continuous increase in *V*_*oc*_ with increasing plasmonic NPs doping, attributed to an upward shift of the fermi level of the photoanode, as a result of the electron-sink effect of charge accumulation by the Ag core NPs, and/or a Bursten-Moss shift effect, from abundantly generated charges. This rising fermi level would lead to poor charge injection efficiency, and hence would account for the decreasing *J*_*sc*_ values in the higher doped plasmonic samples. However, at 1% doping a slight increase in *J*_*sc*_ is observed, which is attributed to the positive plasmonic NPs effects on electron kinetics. Thus, although at this relatively higher percentage of plasmonic doping with resultant high light absorbance, Fig. 3(b), the poor charge injection efficiency due to the rising fermi level, would lead to lower *J*_*sc*_. Nevertheless, we predicted that due to the electron-sink effect of the core Ag NPs, observed in Fig. 3(a), we expected a reduction in charge recombination and improved charge transport, evidenced in the improving *FF* value with increasing plasmonic NPs doping. Thus, this would account for the slight increase in *J*_*sc*_; an optimal concentration for the balance of the negative and positive effects of the plasmonic NPs. In Fig. 5(e) and Table 1, there is a decrease in FF at 0.1% doping, this is attributed to exponential charge recombination due to either high density of charge carriers in the CB of the photoanode TiO_2_ NPs^38^ and/or closeness of the CB edge (fermi level) of the TiO_2_ to the redox.

potential, favoring easy charge transfer to the I^-^/I_3_^-^ redox couple^28^.

The PCE is described as = (*J*_*sc*_ * *V*_*oc*_ * *FF*) / *P*_*in*_; where *P*_*in*_ is the incident light power per unit area. Thus, the observed variations in the PCEs are from the variations in the values of *J*_*sc*_, *V*_*oc*_ & *FF*, which have been discussed above, and hence, explains the analogous PCE results trend observed in Fig. 5(f) and Table 1.

However, of noteworthy is that although sample 0.1% generated the highest *J*_*sc*_ (due to, mainly, efficient charge injection), it is sample 1%, that records the highest PCE, mainly from the enhanced charge transport kinetics and the optimal balancing of the negative and positive effects of plasmonic NPs on the performance of DSSCs. This is interesting because it would suggest that tuning plasmonic NPs incorporation into DSSCs for efficient light harvesting should not be the only desired effect for enhanced DSSC PCE, but also tuning for the optimal balancing of the positive and negative effects of plasmonic NPs can also achieve enhanced PCE, and should be considered in plasmonic DSSCs.

We also measured the I-V characteristics of the samples 5 days after the first measurement of the prepared DSSCs to study the protective effect of the TiO_2_ shell against the corrosive electrolyte in the complete DSSCs. The results for the key parameters are shown in Table 2. As can be observed there is no significant difference between the first measurement and the measurement after 5 days, but with the maintenance of the plasmonic effect trend. Thus, our thin TiO_2_ shell coating served as an effective protection of the plasmonic Ag core against the corrosive electrolyte. This is significant because other works have suggested the need for thicker shells for effective protection at the expense of significantly reducing the LSPR reach, or the use of complex composite shells coatings^29–31^. This may be due to a very compact layering of the thin shell.

**Table 2:** Key I-V characterization parameters to show plasmonic DSSC stability after 5 days.

**Sample**	***J***_***SC***_ **(mA cm** ^**-2**^ **)**	***V***_***OC***_ **(V)**	***FF***	**PCE (%)**
**0%**	9.76	0.714	0.530	4.70
**After 5 days**	9.74	0.709	0.531	4.66
**0.1%**	10.90	0.696	0.501	4.84
**After 5 days**	10.89	0.702	0.502	4.88
**0.25%**	9.00	0.719	0.549	4.52
**After 5 days**	9.04	0.722	0.546	4.54
**0.5%**	8.42	0.724	0.537	4.17
**After 5 days**	8.48	0.727	0.547	4.29
**1%**	8.96	0.741	0.589	4.98
**After 5 days**	8.93	0.744	0.591	5.00
**5%**	3.80	0.774	0.640	2.39
**After 5 days**	3.78	0.777	0.641	2.40

**Figure 6:**
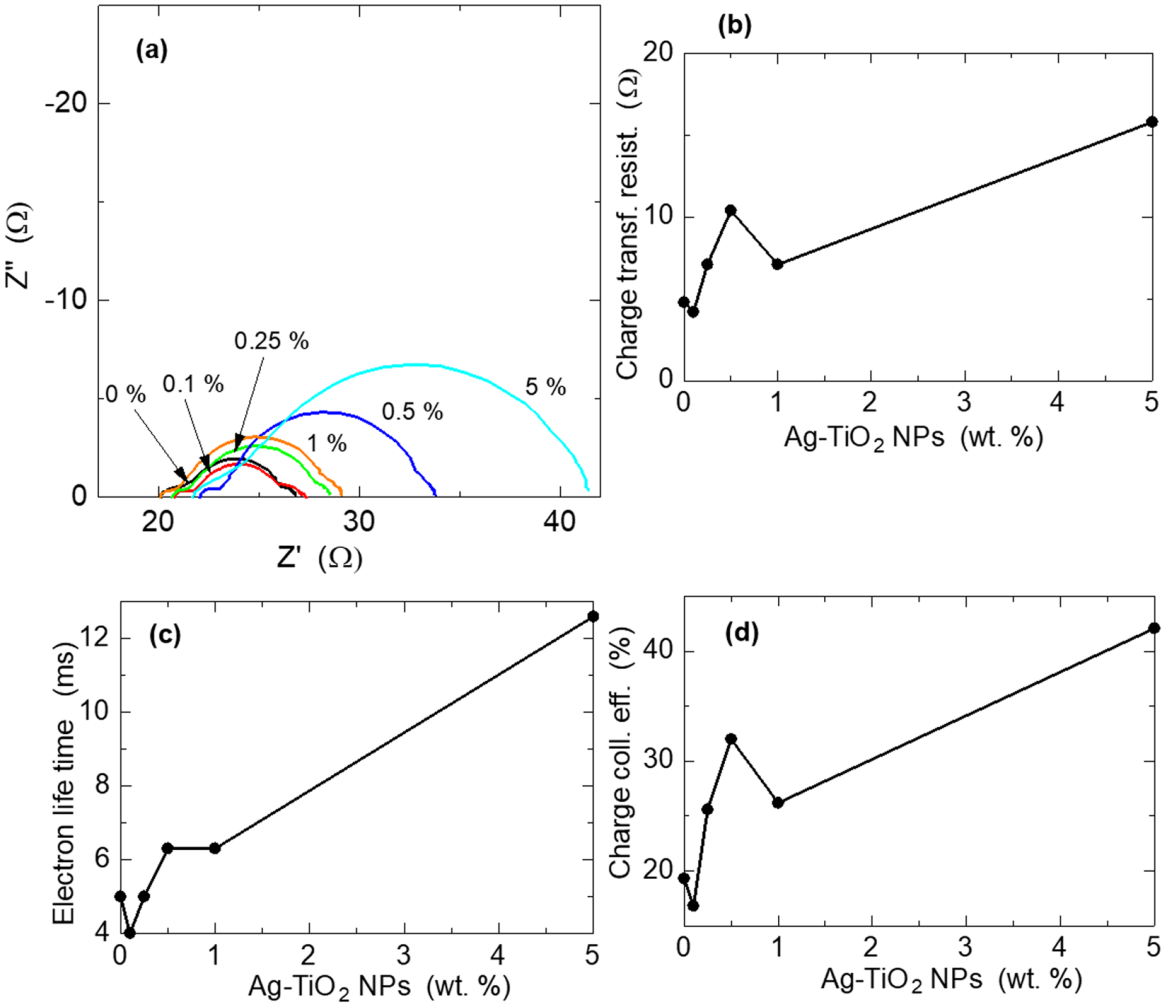
Extracted parameter plots from EIS measurement of DSSCs: (**a**) Nyquist plots. Change in: (**b**) charge transfer resistance, (**c**) electron lifetime and (**d**) charge collection efficiency, with respect to amount of plasmonic NPs doping.

The I-V characterization technique reveals the performance of the solar cell but does not explain the “why” of that performance. EIS is one of the characterization techniques used to study the electron kinetics and charge recombination within the cell, which are responsible for the PCE of the cell. Thus, we evaluated the EIS characteristics of our DSSCs to ascertain the effect of the plasmonic NPs on the electron kinetics of the cells that resulted in the observed I-V characteristics. The results are shown in Table 1 and Fig. 6.

Figure 6(a) is the Nyquist plots of the DSSCs. Basically, there are three semicircles in the Nyquist plots of DSSCs^32^ as observed in our results in Fig. 6(a). These represent the charge transfer kinetics at the counter electrode, photoanode and electrolyte, corresponding to the high (left), mid and low frequency regions, respectively^33–35^. Our DSSC system has the mid-frequency region at 2.51–631 Hz. From Fig. 6(a) it can be seen that there is no significant difference in the two extreme semicircles (electron kinetics at the counter electrode and I^−^/I_3_^−^ species diffusion in the electrolyte) of the DSSC samples, indicating that the plasmonic NPs do not affect the charge kinetics of these components of the DSSCs. In addition, the intercept of the Nyquist plots of the DSSCs at the Z′-axis, at the high frequency end, are almost the same, fluctuating randomly in the range of 20–22 Ω. This intercept point represents the series resistance, *R*_*s*_, of the cells, arising from the FTO substrates and the external circuit wires. Thus, their approximately constant value among the samples is understandable because the plasmonic NPs are not in direct contact with these components, and thus, not expected to affect them. However, Lim *et al*.^21^. reported varying *R*_*s*_ in different Ag NPs-doped DSSC samples. This could be due to their use of bare Ag NPs and relatively high amounts of doping (5–40%). From Fig. 6(a), the diameter of the mid-semicircle, generally, increases with increasing amount of plasmonic NPs. This diameter represents the charge transfer resistance against recombination, *R*_*ct*_, at the TiO_2_-Dye/electrolyte interface. The plasmonic effect trend on this parameter is shown in Fig. 6(b) and Table 1. This general trend of increasing *R*_*ct*_ with increasing amount of plasmonic NPs is attributed to the electron-sink effect, which reduces the amount of electrons recombining with electrolyte hole species at the interface. Thus, the electron-sink effect has both a negative and positive effect of reducing charge injection efficiency via upward shift of fermi level and reduction of charge recombination via electron capture, respectively. The two deviations from the general effect trend at 0.1 and 1% dopings are attributed to increased charge recombination from high charge densities in the CB of TiO_2_, as discussed above. Figure 6(c) shows the effect of the plasmonic NPs on electron lifetime of charge carriers, *τ*_*n*_, a measure of charge recombination. It, generally, increased with increasing plasmonic NPs content, attributed to the electron-sink effect of capturing excited charges by the core Ag NPs. Thus, these plasmonic NPs improved the lifetime of excited charge carriers to improve the recombination characteristics of the cells, as observed in the *R*_*ct*_ results. The drop in *τ*_*n*_ at 0.1% is attributed to the increased recombination as a result of high charge carrier density, in addition to the relatively low amount of plasmonic NPs doping. At 1% doping, although there is an enhancement of *τ*_*n*_, the lack of the anticipated increase in value is also attributed to increased recombination as a result of high electron density in TiO_2_. The electron lifetime values were obtained from the relation, *τ*_*n*_ = 1 / (2π*f*)^36^, where *f* is the frequency at the peak point of the mid-frequency semicircle. We also extracted the charge collection efficiency, *η*_*cc*_, to investigate how efficiently the plasmonic NPs affect charge carriers transported from the conduction band of TiO_2_ to the back contact FTO. The results are shown in Fig. 6(d) and Table 1. The values were obtained using the relation, 1 / [1 + (*R*_*s*_ / *R*_*ct*_)]^36^. Generally, the trend is that, *η*_*cc*_ enhanced with an increase in amount of plasmonic NPs. This is also attributed to the electron-sink effect of the core Ag NPs capturing electrons and protecting them from recombination. These captured electrons are released subsequently, in an analogous fashion as the trapping-detrapping electron diffusion transport model of DSSCs. However, there is a deviation to the increasing *η*_*cc*_ trend at samples 0.1% and 1% by their lower values, compared to those of their immediate preceding samples. These deviations are attributed to the exponential charge recombination in these samples (confirmed in their *R*_*ct*_ and *τ*_*n*_ values), as a result of the high charge density in the TiO_2_ NPs of these samples. It is also interesting to note that these samples have the lowest *η*_*cc*_s, yet, the highest *J*_*sc*_s, compared to the rest of the samples. This further supports their possession of high charge densities.

**Figure 7:**
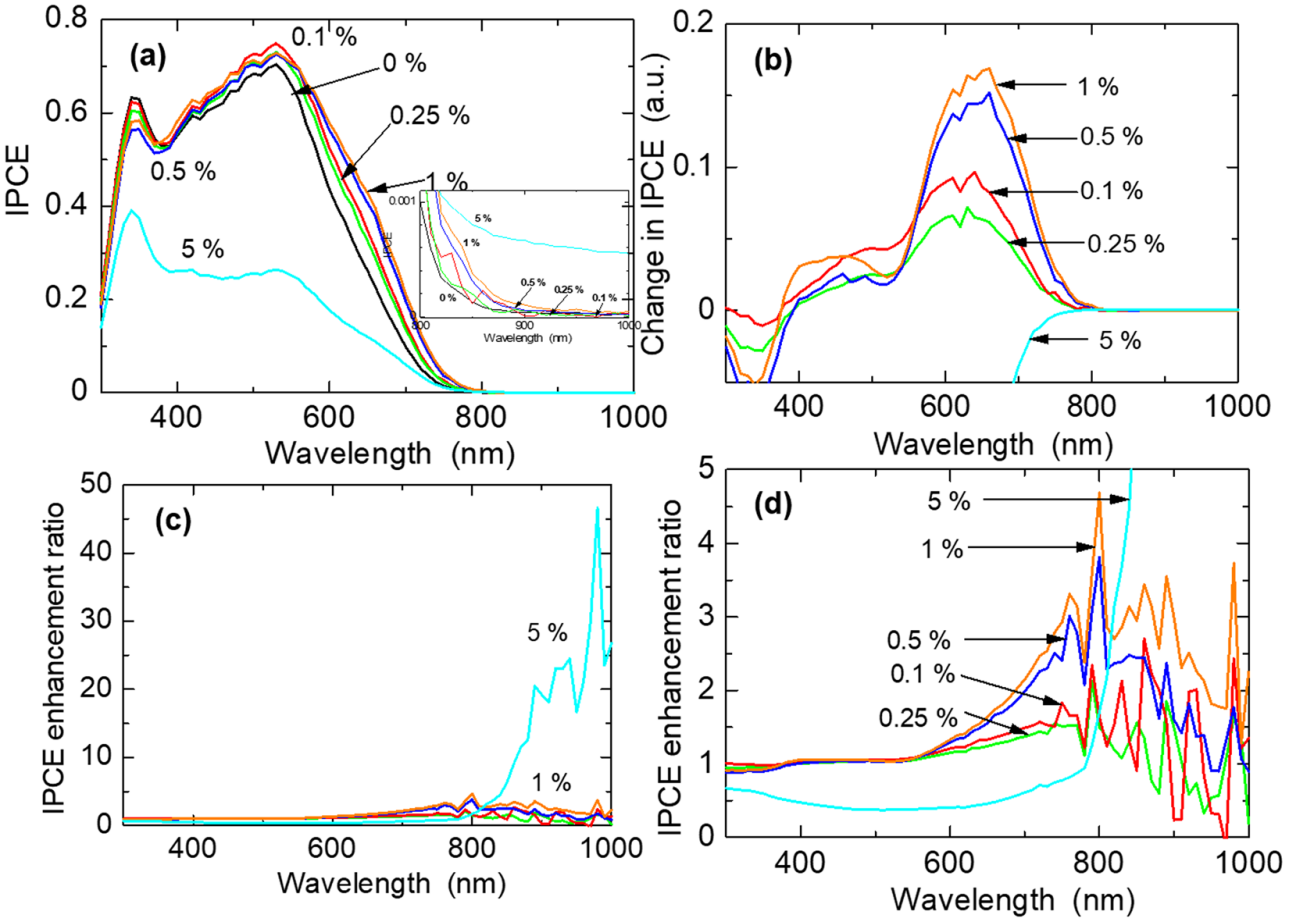
(**a**) IPCE spectra of the fabricated DSSCs to show the effect of plasmonic NPs on device performance with respect to wavelength, inset: expanded NIR region to show IPCE activity; (**b**) the change in IPCE of plasmonic NPs-doped DSSCs over the pristine DSSC; (**c**) the relative change in IPCE of plasmonic NPs-doped DSSCs over the pristine DSSC; (**d**) an expanded part of figure (**c**).

In order to investigate the effect of the plasmonic NPs on the cell performance at each wavelength, we evaluated their IPCE performance which is shown in Fig. 7. Results in Fig. 7(a) show an analogous trend as observed in the I-V characterization performance, and hence, have the same explanations. However, in the UV region, there is a general trend of reducing performance with increasing plasmonic NPs content. This observation is clearly seen in Fig. 7(b), obtained from the deduction of the IPCE of the reference cell from those of the plasmonic cells. It shows that the reference pristine cell performs better than the plasmonic DSSCs. This is attributed to the reduction in the effective number of TiO_2_ P25 NPs replaced by the Ag in the photoanode as a result of the higher relative atomic mass of Ag than the molecular mass of TiO_2_. It also indicates that the plasmonic effect of these NPs does not enhance cell performance in the UV region. Figure 7(b) shows that the plasmonic NPs enhance cell performance across the visible and NIR regions, confirming the broadband enhancement effect of our Ag@TiO_2_ C-S NPs. However, the highest region of the plasmonic enhancement effect is in the range of 550–720 nm. For the 5%-doped cell, there is no IPCE enhancement effect from 300–720 nm, which is the active light absorption region of the N719 dye.

The IPCE can also be described as, IPCE = *η*_*lh*_(λ) * *η*_*inj*_(λ) * *η*_*reg*_(λ) * *η*_*cc*_(λ); where, *η*_*lh*_(λ) is light harvest efficiency, *η*_*inj*_(λ) is charge injection efficiency and *η*_*reg*_(λ) is dye regeneration efficiency. Thus, the non-performance in this region is due to poor charge injection, and/or probably, in addition to poor dye regeneration. However, it clearly indicates that the plasmonic NPs are behaving as direct sensitizers in the NIR region. We further investigated the relative IPCE enhancement effect of the plasmonic NPs by dividing the IPCEs of the plasmonic cells by that of the reference cell. The results are shown in Fig. 7(c), with the expanded form shown in Fig. 7(d). There is a reversal of the plasmonic NPs enhancement effect on IPCE performance trend, in an observed increasing IPCE enhancement effect with increasing plasmonic NPs content.

## Conclusion

We have successfully prepared Ag@TiO_2_ NPs with undulating shell structure, to study the effects of plasmonic NPs on the performance of DSSCs via extended characterization and a systematic approach. These plasmonic NPs had a broadband enhancement effect in their DSSCs. They enhanced cell performance via four main routes: 1) enhancement of light absorption by the N719 dye via their LSPR effect; 2) modification of the fermi level of the TiO_2_ photoanode for efficient charge injection; 3) enhancement in the NIR region by acting as direct sensitizers; and 4) balancing the negative and positive electron-sink effects of the core Ag NPs, at optimal doping content, on cell performance parameters of *J*_*sc*_, *V*_*oc*_, *FF*, *R*_*ct*_, and *τ*_*n*_. Two optimal doping concentrations were observed with enhanced PCE over a reference pristine cell of PCE of 4.66%: 1) 0.1%; with the highest *J*_*sc*_, with the main enhancement via efficient charge injection, by lowering the fermi level of the TiO_2_ photoanode, producing a PCE of 4.88%; and 2) 1%; with the main enhancement via the electron-sink effect improving cell performance parameters of *J*_*sc*_, *V*_*oc*_, *FF* and *R*_*ct*_, producing a PCE of 5.00%. These two samples produced the highest *J*_*sc*_s, respectively, although on the other hand they recorded the lowest *η*_*cc*_s. Thus, the plasmonic NPs exhibited the anticipated characteristics for highly efficient DSSCs, however, the intrinsic exponential charge recombination at high charge carrier density on TiO_2_ prevented the realization of making the expected high impact. Nevertheless, with the appropriate modification to harvest the abundant charge carriers generated, this expected impact for highly efficient DSSCs can be achieved. To put it rather simply, if a plasmonic DSSC is designed to harvest the high charge density of electrons induced by the plasmonic NPs, then extremely high PCE can be achieved to meet the desired high expectation of plasmonic DSSCs. In addition, if the fermi level upward shift effect is controlled, then higher plasmonic NPs loaded DSSCs can be prepared, without significant loss to cell performance to inefficient charge injection. This would also increase the cell performance in the energy-rich infrared region of the solar energy flux. Thus, we believe our results might serve as invaluable guide for the preparation of highly efficient plasmonic DSSCs. However, our work is based on just one plasmonic nanostructure, and since the LSPR effect is dependent on the composition, size, environment and shape of the plasmonic NP. Thus, further studies based on these properties would give a deeper and wider understanding of the effects of plasmonic NPs in DSSCs.

## Methods

### Synthesis of Ag@TiO_2_ NPs

The C-S NPs were prepared using a facile solution process with Pluronic P123 as a dispersing agent, in an air-tight 1 L polyethylene terephthalate container covered with an Al foil. In a typical reaction 3.11 g of Pluronic P-123 (Aldrich; PEG-PPG-PEG, avg. M_n_ ~ 5800) was dissolved in 215.25 ml 1-butanol (Wako; dehydrated, 99.0%) by stirring on a hot plate with a magnetic stirrer, at ambient temperature, at 500 rpm for 10 min. A 3.585 ml of 0.5 mol L^−1^ AgNO_3_ (Wako; 99.8 wt.%) was added, in drops, with continuous stirring for another 1 h. The solution was sonicated for about 5 min. and 3.585 ml of 1 mmol L^−1^ of L( + )-Ascorbic acid (Wako; 99.6 wt.%) was added with continuous stirring for another 1 h. A second solution of made of 1.02 g of titanium tetraisopropoxide (Wako; 95.0%) in 322.5 ml 1-butanol, chelated with 0.234 ml acetylacetone (Wako; 99.0 wt.%) in 18 ml 1-butanol, was added and stirred for a further10 min. An extra solution of 3.75 g of Pluronic P-123 in 37.5 ml 1-butanol was then added and stirred for 20 min., with a subsequent 5 min. sonication. The temperature of the hot plate was set to 70 °C and the reaction mixture stirred at this temperature for 1 h. After this, Ag@TiO_2_ NPs sol was aged at 60 °C for 1 week, by which time the C-S NPs had settled at the bottom of the container. The supernatant solution was decanted off and the residue heat-treated at 80 °C into a gel. The gel residue was further heat-treated at 350 °C for 1 h, based on thermogravimetric-deferential thermal analysis (TG-DTA) results, to obtain the pure Ag@TiO_2_ NPs. This is the result of an optimized synthesis process to obtained a desired ~ 20 nm size C-S NPs.

### Pastes and photoanodes preparation

The paste for the photoanodes fabrication were prepared using a planetary ball miller (Fritsch pulverisette 7) with alumina mortar and balls. For the pristine, n-doped, paste (0%), 1.74 ml of ethylene glycol (dehydrated, Wako; 99.5% min.) was added to 1.0 g of TiO_2_ P25 (Sigma-Aldrich; 21 nm; ≥99.5%) and then milled at 500 rpm for 1 hr. After that 1.69 g Citric acid (Chameleon reagent; anhydrous, ≥99.5%) was added and milled again for another 1 h. For the Ag@TiO_2_ C-S NPs-doped pastes, the same preparation procedure was used with the appropriate mass of Ag@TiO_2_ replacing the appropriate amount of TiO_2_ P25 to obtain the doped pastes (0.1, 0.25, 0.5, 1 & 5%).

The photoanodes were prepared via a doctor blade coating on fluorine-doped tin oxide (FTO)-on-glass substrates (85% T, 9 Ω/□; 3 × 2 cm). The FTO substrates were first cleaned by RCA treatment and coated with a TiO_2_ buffer layer via treatment in 0.04 mol L^−1^ TiCl_4_ (Wako; 99.0% min.) solution at 70 °C for 30 min (with 2 cm^2^ active area masking), washed with water and heat-treated at 450 °C for 1 h. The pastes were coated on the FTO substrates using 60 μm scotch tape masks with a circular active area of 0.785 cm^2^ (10 mm diameter) each. For uniformity and reproducibility, the doctor blade was kept at a constant distance over the substrate and an 80 g weight of a 5 × 5 cm steel blocked applied on it to make contact with the substrate. The blade was drawn at a rate of 2.5 mm s^−1^ to spread the paste. The samples were dried sequentially on a hot plate at ambient temperature, 60 °C &120 °C, for 5 minutes each, and finally heat-treated at 500 °C for 1 h. The calcined samples were washed with ethanol and dried with a hot blower. For dye sensitization, the dried samples were immersed in a 0.3 mmol L^−1^ of N719 dye (ALDRICH; 65 mol% dioxole), in a 1:1 solvent system of acetonitrile (Wako; 99.5 m/m%)-tert-butyl alcohol (Wako; 99.0%), for 24 h. The dye-sensitized photoanodes were rinsed with acetonitrile to remove excess dye, and dried using a hot blower.

### Assembling of DSSCs

Sandwich-type cells were prepared using a 50 μm plastic spacer (DuPont, Himilan) with an open area of 1.13 cm^2^ (12 mm diameter). The dye-sensitized photoanodes were sealed to platinized counter electrodes by heating on a hot plate at 105 °C, with a 1 kg weight load, for about 5 min. The counter electrodes were prepared using RCA-cleaned FTO substrates with drilled holes, 12 mm apart, with Pt coating using a sputter coater (Hitachi E-1030 ion sputter) at 15 mA for 600 s. An acetonitrile solvent based electrolyte, composed of 0.05 mol L^−1^ iodine (Aldrich; ≥99%), 0.1 mol L^−1^ lithium iodide (Strem Chemicals; anhydrous, 98% min.), 0.6 mol L^−1^ 1, 2-dimethyl-3-propylimidazolium iodide (TCI; >98%), and 0.5 mol L^−1^ 4-tert-butylpyridine (Aldrich; 96%), was injected into the cell and sealed with a piece of the spacer and scotch tape. DSSCs with Ag@TiO_2_ plasmonic NPs doping concentrations of 0.1, 0.25, 0.5, 1 & 5%, in addition to a reference un-doped DSSC, 0%, were obtained.

### Characterization

The optical absorption characteristics of the plasmonic NPs and photoanodes were evaluated using a JASCO V-670 UV-Vis-NIR spectrophotometer. The particle size, size distribution and core-shell structure of the Ag@TiO_2_ C-S NPs were observed using a JEOL JEM-2100 F TEM. A Rigaku Ultima IV R285S XRD was used to study the composition and crystallinity of the Ag@TiO_2_ C-S NPs. PL spectroscopy of the photoanodes was evaluated using a He-Cd laser (325 nm) as the excitation source. The luminescence was evaluated by a multi-channel CCD spectrometer. The PL properties were measured using a single monochromator containing a grating and a CCD-array. The I-V characteristics of the cells were evaluated using ADCMT 6244 DC Voltage/Current Source/Monitor and an HAL-320 W solar simulator (Asahi spectra) with a 300 W xenon lamp and an air-mass 1.5 global filter. The solar simulator was calibrated to an intensity of 100 mW/cm^2^ (1 sun), using a 1 SUN checker (Asahi CS-40). The IPCE characterization was done via a DC method, using Bunkoukeiki SM-250KB spectrometer with a Keithley 2401 source meter with an irradiation flux of 2.0 × 10^15^ photons, with masking active area of 1 cm^2^. A Hitachi S-4800 FE-SEM was used to study the morphology and thickness of photoanode coatings. The electron transport dynamics of the photogenerated electrons was evaluated via an EIS equipment (Solartron SI 1287 Electrochemical Interface and Solartron1255B Frequency Response Analyzer) under bias of the open circuit voltage, V_oc_, within a frequency range of 0.1 Hz to 1 MHz, and an ac amplitude of 40 mV; and under a light irradiation of 100 mW/cm^2^ ^37,38^.
